# The *RUBY* reporter for visual selection in soybean genome editing

**DOI:** 10.1007/s42994-024-00148-6

**Published:** 2024-03-18

**Authors:** Li Chen, Yupeng Cai, Xiaoqian Liu, Weiwei Yao, Shuiqing Wu, Wensheng Hou

**Affiliations:** 1 State Key Laboratory of Crop Gene Resources and Breeding, Institute of Crop Sciences, Chinese Academy of Agricultural Sciences, Beijing, 100081 China; 2 Ministry of Agriculture Key Laboratory of Soybean Biology (Beijing), Institute of Crop Sciences, Chinese Academy of Agricultural Sciences, Beijing, 100081 China

**Keywords:** Soybean, RUBY, Visual reporter, Genome editing, Biotechnology

## Abstract

Current systems to screen for transgenic soybeans (*Glycine max*) involve laborious molecular assays or the expression of fluorescent markers that are difficult to see in soybean plants. Therefore, a visual system for early screening of transgenic plants would increase the efficiency of crop improvement by genome editing. The *RUBY* reporter system, which consists of three genes encoding betalain biosynthetic enzymes, leading to the accumulation of purple pigment in transgenic tissue, has been employed in some plants and dikaryon fungi. Here, we assessed the *RUBY* reporter for visual verification during soybean transformation. We show that *RUBY* can be expressed in soybean, allowing for visual confirmation of transgenic events without the need for specialized equipment. Plants with visible accumulation of purple pigment in any tissue were successfully transformed, confirming the accuracy of the *RUBY* system as a visual indicator. We also assessed the genetic stability of the transgene across generations, which can be performed very early, using the cotyledons of the progeny. Transgene-free seedlings have a distinct green color, facilitating the selection of genome-edited but transgene-free soybean seedlings for harvest. Using the *RUBY* system, we quickly identified a transgene-free *Gmwaxy* mutant in the T1 generation. This system thus provides an efficient and convenient tool for soybean genome editing.

Dear Editor,

Recent advancements in genome editing have contributed to crop improvement by enabling faster, cheaper, and more precise modifications of the plant genome. However, employing genome-editing systems such as clustered regularly interspaced short palindromic repeat (CRISPR)/CRISPR-associated nuclease 9 (Cas9) using Agrobacterium (*Agrobacterium tumefaciens*)–mediated transformation (Zlobin et al. 2020) requires efficient methods to transform and identify plants carrying the transgene. Moreover, once the CRISPR/Cas9 system has edited the target gene, producing a transgene-free commercial product requires the removal of the transgene by backcrossing and genetic segregation.

Transformation of soybean (*Glycine max*) entails regeneration via organogenesis from cotyledons incubated with Agrobacterium cultures harboring the transgene of interest (Legendre and Demirer 2023). New shoots develop from these cotyledon nodes, and some of these shoots carry the transgene. Many transgenic plants may be produced at one time, making assays to determine transformation efficiency costly and time-consuming. It is vital to identify primary transgenic (T0) plants at this initial stage, as the T0 plants can then be genotyped for the presence of the desired gene. Hence, a visual system for the early screening of transgenic plants would transform soybean cultivation, allowing for increased efficiency and decreasing our dependency on traditional assays, such as PCR.

Visual assessments of transgenic events have extensively employed green fluorescent protein (GFP), β-glucuronidase (GUS), or the red fluorescent protein DsRed2, encoded by the transgene-carrying genome-editing reagents in rice, maize, and other plants. Despite much effort, we found it challenging to detect GFP or DsRed2 fluorescence in soybean tissues. Notably, the *RUBY* reporter system consisting of three genes encoding betalain biosynthetic enzymes has been used to visualize transgenic events in Arabidopsis (*Arabidopsis thaliana*), rice, and cotton (*Gossypium hirsutum*) (Polturak et al. 2016; He et al. 2020; Ge et al. 2023; Li et al. 2023). *RUBY* leads to the accumulation of betalain pigment in transgenic tissue, thereby providing a visual marker for transformation. *RUBY* was recently used during in vitro and *ex vitro* hairy root transformation of soybean (Niazian et al. 2023). Based on this report, we hypothesized that *RUBY* would be useful to identify transgenic soybean plants, providing a non-invasive, rapid, and cost-effective indicator of transformation and presence of the transgene.

Here, we constructed a new CRISPR/Cas9 vector with a *RUBY* cassette for visual verification (Fig. 1A) and targeted *GmWaxy* (Glyma.10G172200). Following Agrobacterium infection, we detected the red pigment in the cotyledon explants after 3 days of co-cultivation. The regenerated plants showed dark purple coloration in their stems, leaves, and roots during the selection, elongation, and rooting stages, respectively (Fig. 1B). Thus, *RUBY* can be expressed in soybean, allowing for simple visual inspection.

**Fig. 1:**
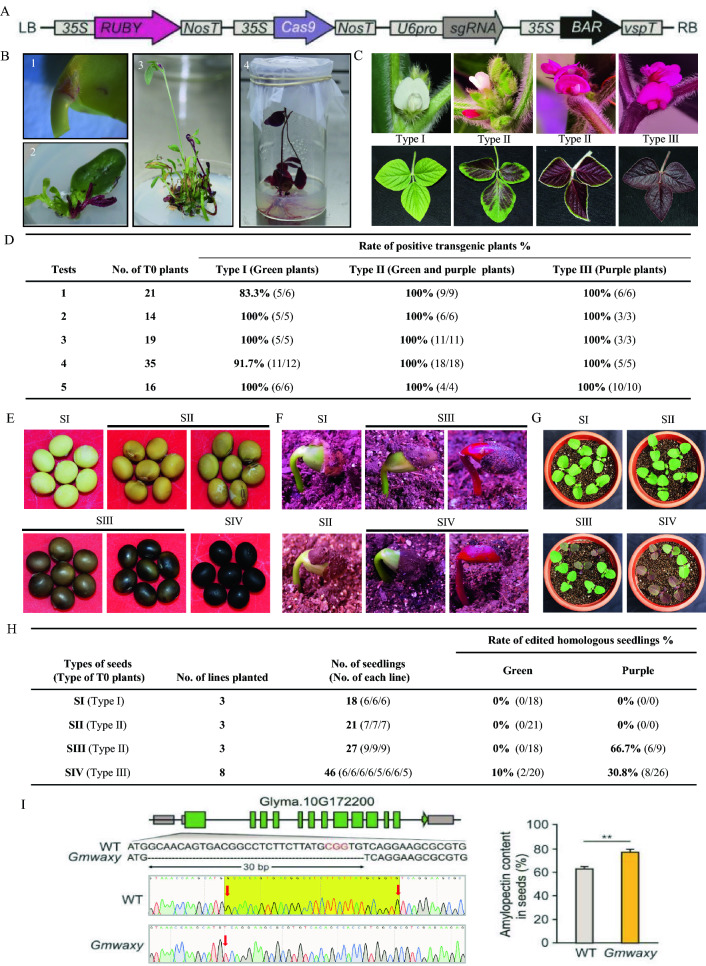
A *RUBY* reporter system applied to genome editing in soybean. **A** Diagram of the *RUBY* reporter construct included in the CRISPR/Cas9 vector. LB, left border; RB, right border. **B** Betalain accumulation during the co-cultivation stage (1), selection stage (2), elongation stage (3), and rooting stage (4) of soybean transformation. **C** Representative photographs of the three types of primary transgenic plants obtained. **D** Summary of the rate of positive transgenic plants among the three types of primary transgenic plants. **E** Representative photographs of the four types of T1 seeds obtained from T0 transgenic plants. **F** T1 progeny at the VE (cotyledons above the soil surface) seedling stage from different types of seeds harvested from primary transformants. **G** T1 progeny at the VC (expanded unifoliolate leaves) seedling stage from different types of seeds. **H** Summary of the inheritance pattern and gene editing rate in T1 seedlings derived from seeds of different groups. **I** Diagram of the *GmWaxy* locus showing the location of the sgRNA target site and sequence chromatogram for the wild type (WT) and a homozygous *Gmwaxy* mutant by CRISPR/Cas9 (left) and associated amylopectin content in the WT and *Gmwaxy* (right). **P < 0.01, as determined by analysis of variance (ANOVA)

We observed a range of betalain accumulation among plants, from no visible pigment to dark purple flowers and leaves, likely reflecting the expression levels of *RUBY*. We divided all putative primary transformants into three groups: plants with green stems, green leaves, and white flowers (Type I); plants with green and/or purple stems, green and purple leaves, and white and/or purple flowers (Type II); and plants with purple stems, leaves, and flowers (Type III) (Fig. 1C). We genotyped T0 plants from five independent tests by PCR and polyacrylamide test (PAT) strips, revealing that 100% of Type II and III plants carried the transgene, while 83.3–100% of Type I plants did (Fig. 1D). We conclude that in all cases, a plant with visible purple coloration in any tissue is a successful transformation event.

Using the reporter to track the presence of the transgene requires stable expression across generations. To assess the stability of transgene expression between generations, we harvested mature seeds from each type of plant and scored their color: yellow (SI), brown (II), aubergine and brown (SIII), and dark aubergine (SIV) (Fig. 1E). Type I plants only produced SI seeds, Type II plants produced seeds of all colors (SI–SIV), and Type III plants produced only SIV seeds. When we sowed each seed type, we determined that seedlings harboring the transgene can be identified by their cotyledon color as early as the VE stage when cotyledons emerge above the soil surface (Fig. 1F). The SI and SII seeds gave rise to only green seedlings, whereas the SIII and SIV seeds germinated into both green and purple seedlings (Fig. 1F, G). We selected three lines each from the SI and SII groups; all seedlings were green and non-edited. In the SIII group, 18 out of 27 seedlings from three selected lines were green and harbored the wild-type sequence at *GmWaxy*, whereas the remaining nine seedlings were purple, PAT positive, and edited. Of these, 66.7% (6/9) purple seedlings carried a homozygous editing event at the *GmWaxy* locus. From the SIV group, 20 out of 46 seedlings were green, of which 10% (2/20) were nevertheless homozygous and edited at *GmWaxy*. The remaining 26 seedlings were purple and PAT positive, of which 30.8% (8/26) were homozygous and edited at *GmWaxy*. The SIII and SIV groups therefore contained purple and green T1 seedlings with a homozygous mutation in *GmWaxy* (Fig. 1H). Dark purple T0 seedlings should thus be selected and their seeds collected; the absence of the transgene can be ascertained early in the cotyledon stage of the offspring, enabling the quick identification of transgene-free seedlings based on their distinct green color.

With this reporter system, we identified a transgene-free *Gmwaxy* mutant harboring a 30-bp deletion in the T1 generation (Fig. 1I). This mutant showed an amylopectin content of 77.5% in seeds, which was significantly higher than in the wild type (63.5%) (Fig. 1I).

In conclusion, we applied the *RUBY* system for stable soybean transformation and determined that it is an effective, non-invasive, affordable system for selecting successful transformation events and transgene-free plants. In previous studies, similar reporters have enabled visual identification of haploids in maize and tomato (*Solanum lycopersicum*) (Wang et al. 2023), suggesting the potential for adoption of this reporter in soybean haploid breeding. The *RUBY* system provides an efficient and convenient tool for soybean editing, with many potential applications in basic research and biotechnology.

## Materials and methods

### Vector construction and soybean transformation

The *CYP76AD1*, *DODA*, and *Glu-T* coding sequences form the *RUBY* system genes are expressed as a polycistronic transcript under the control of the cauliflower mosaic virus (CaMV) 35S promoter. A single guide RNA (sgRNA) was designed according to the CRISPR-P website (http://cbi.hzau.edu.cn/cgi-bin/CRISPR) to target *GmWaxy* via CRISPR/Cas9-mediated gene editing. The target site was 5′-AGTGACGGCCTCTTCTTATG-3′. The soybean (*Glycine max* L.) cultivar ‘Jack’, with green leaves, white flowers, and the yellow seed coat, was used as the recipient for transformation, using an *Agrobacterium tumefaciens*–mediated transformation method as described previously (Chen et al. 2018).

### DNA extraction and PCR detection

Genomic DNA was extracted from the leaves. Genotyping PCR was performed using Phanta Super Fidelity DNA Polymerase with the *GmWaxy* forward primer (5′-CGCAAGCTTCTGGTAGAACA-3′) and reverse primer (5′-ATCCACATACAGCCAACGCT-3′). The presence of the *bar* gene in transgenic plants was tested by PCR with the primer pair 5′-GCACCATCGTCAACCACTACATC-3′ (forward) and 5′-CAGAAACCCACGTCATGCCAGTT-3′ (reverse).

### Determination of amylopectin contents

The amylopectin content was determined using an Amylopectin Microplate Assay Kit (cat. CAK1179, Cohesion) and Starch Microplate Assay Kit (cat. CAK1022, Cohesion) according to the manufacturer’s instructions.

## Data Availability

All data generated or analyzed during this study are available from the corresponding author upon reasonable request.
