# Correction: The *RUBY* reporter for visual selection in soybean genome editing

**DOI:** 10.1007/s42994-024-00156-6

**Published:** 2024-10-02

**Authors:** Li Chen, Yupeng Cai, Xiaoqian Liu, Weiwei Yao, Shuiqing Wu, Wensheng Hou

**Affiliations:** 1 State Key Laboratory of Crop Gene Resources and Breeding, Institute of Crop Sciences, Chinese Academy of Agricultural Sciences, Beijing, 100081 China; 2 Ministry of Agriculture Key Laboratory of Soybean Biology (Beijing), Institute of Crop Sciences, Chinese Academy of Agricultural Sciences, Beijing, 100081 China

**Correction: aBIOTECH** 10.1007/s42994-024-00148-6

The copyright holder for this article was incorrectly given as ‘Agricultural Information Institute, Chinese Academy of Agricultural Sciences’ but should have been ‘The Authors’.

This article was originally published with the incorrect licence; it should have been:

**Open Access** This article is licensed under a Creative Commons Attribution 4.0 International License, which permits use, sharing, adaptation, distribution and reproduction in any medium or format, as long as you give appropriate credit to the original author(s) and the source, provide a link to the Creative Commons licence, and indicate if changes were made.

The images or other third party material in this article are included in the article’s Creative Commons licence, unless indicated otherwise in a credit line to the material. If material is not included in the article’s Creative Commons licence and your intended use is not permitted by statutory regulation or exceeds the permitted use, you will need to obtain permission directly from the copyright holder.

To view a copy of this licence, visit http://creativecommons.org/licenses/by/4.0/.

